# Pilocytic Astrocytoma Enlargement Following Irradiation: Relapse or Pseudoprogression?

**DOI:** 10.7759/cureus.1045

**Published:** 2017-02-21

**Authors:** Yury Trunin, Andrey V. Golanov, Valery V Kostjuchenko, Mikhail Galkin, Alexander N Konovalov

**Affiliations:** 1 Radiology and Radiosurgery, Burdenko Neurosurgery Institute; 2 Department of Radiation Oncology, Burdenko Neurosurgery Institute; 3 Gamma Knife, Neurosurgery Business Center; 4 Department of Radiation Oncology, Burdenko Neurosurgical Institute; 5 Burdenko Neurosurgery Institute

**Keywords:** radiotherapy, pseudoprogression, pilocytic astrocytoma, irradiation

## Abstract

Pilocytic astrocytomas are the most common gliomas (World Health Organization (WHO) grade 1) in children. According to many authors, stereotactic irradiation (radiotherapy and radiosurgery) is an effective method that provides a prolonged, recurrence-free survival in patients who have had partial tumor removal or in patients with an unresectable tumor.

We present a case of a patient with midbrain pilocytic astrocytoma that enlarged considerably in the first six months after irradiation and then diminished in size during the next six months without any antitumor treatment. The clinical and radiological follow-up data for this patient, the surgery results for similar patients, and the data from other authors led us to the conclusion that early tumor volume enlargement after irradiation is usually pseudoprogression.

## Introduction

Radiation therapy is an important modality used in the treatment of patients with pilocytic astrocytomas (PA). Stereotactic irradiation is an effective method of PA treatment in patients with residual tumors or relapse, patients with progressive disease after a previous surgery, and patients with unresectable tumors.

Tumor volume enlargement is a possible result of stereotactic irradiation, and a determination of whether the enlargement is a true relapse or pseudoprogression is critical, because each situation requires a different treatment.

## Case presentation

A 20-year-old female patient with a long history of headaches presented to the Burdenko Neurosurgical Institute, Moscow, Russia. Since mid-2008, the frequency and the intensity of her headaches increased along with the presentation of nausea and vomiting in the morning. This initiated our investigation. A magnetic resonance imaging (MRI) showed a midbrain tumor and occlusive hydrocephaly. In August 2008, the patient underwent a surgical resection of the midbrain PA (World Health Organization (WHO) grade I) and endoscopic third ventriculostomy (ETV).

**Figure 1:**
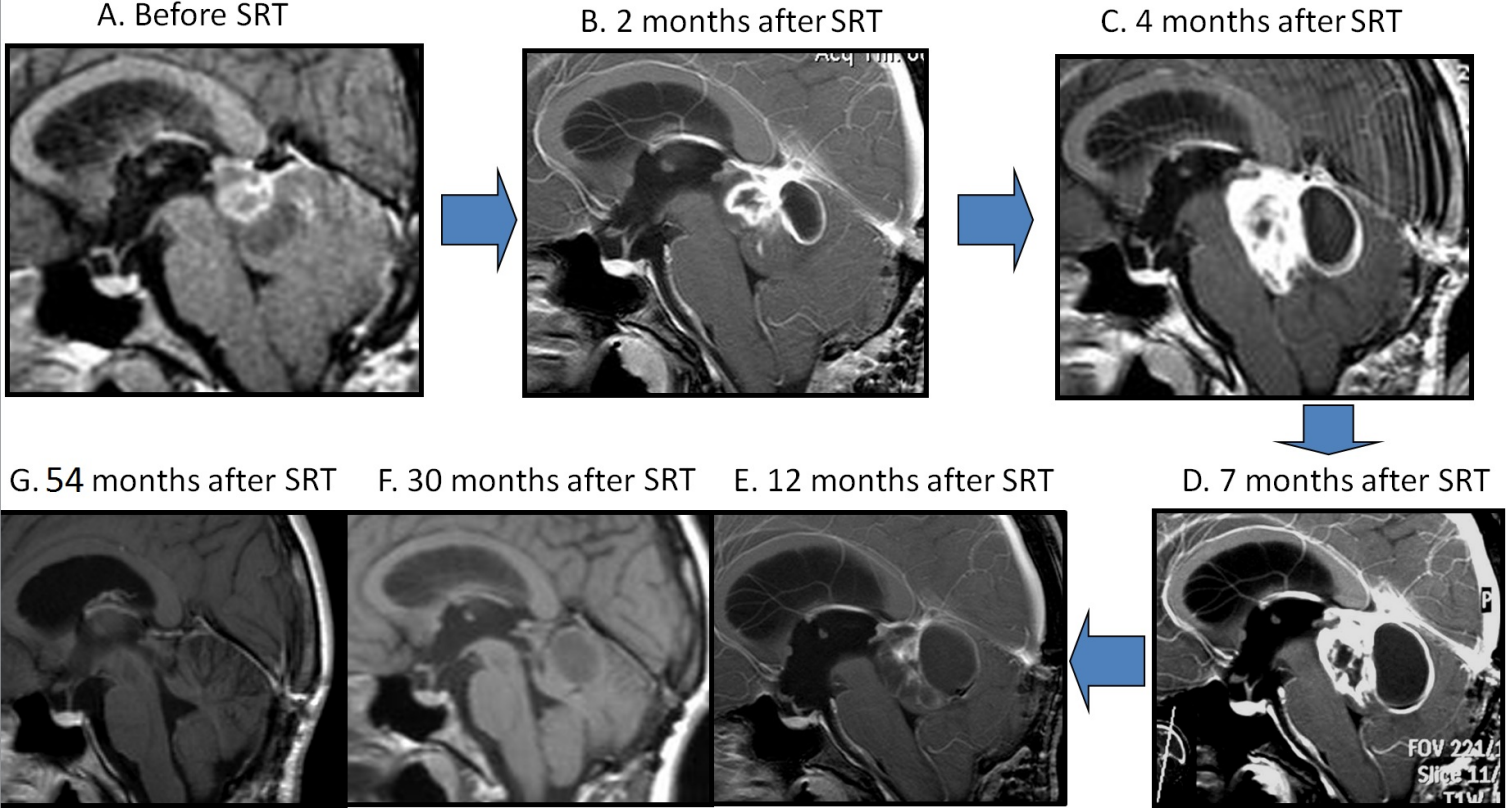
Pilocytic astrocytoma MRI pseudoprogression dynamic after 54 Gy SRT: temporary enlargement during the first year with subsequent regression

At her three-month postsurgery follow-up, an MRI scan showed a residual tumor at the region of the midbrain and sylvian aqueduct. The tumor consisted of cystic and solid components and captured contrast media heterogeneously (see Figure 1A). No neurological signs were found with the investigation.

Taking into account the presence of the residual tumor and its infiltrative growth, stereotactic radiotherapy (SRT) was performed from October through November 2008. A Novalis 6 MV linac with micro-multileaf collimator (Brainlab, Munich, Germany) was used. Gross tumor volume (GTV) included changes at T2 MRI sequence together with the region of contrast enhancement. Clinical target volume (CTV) was taken as GTV plus a 5 mm margin, planning target volume (PTV) was taken as CTV plus a 2 mm margin and reached 36.2 cm^3^. We used the standard fractionation scheme: 1.8 Gy per fraction up to a total margin dose of 54 Gy (at the 90% isodose line). The maximum dose was 61.8 Gy, the minimum dose was 54 Gy, and the median dose was 60 Gy. The dose was delivered with three dynamic conformal arcs. The patient state was stable during the course of treatment.

The first follow-up MRI was performed two months after the SRT and showed tumor enlargement in both cystic and solid components with intense contrast enhancement (see Figure 1B). The patient status was stable, which prompted us to continue observation along with a two-week course of steroids.

The investigations at four and seven months after the SRT showed tumor enlargement without any clinical deterioration (see Figures 1C-1D). The sylvian aqueduct was still blocked, but due to the ETV, no signs of hydrocephaly appeared. Positron emission tomography (PET) with 11C-methionine showed an increased tracer uptake (tumor/non-tumor ratio = 1.45). The leading neurosurgeons of our institute proposed a repeated tumor resection, but the patient rejected the operation given the absence of deterioration and an assurance of relapse.

An MRI 12 months after the SRT showed a decrease of the tumor volume and contrast enhancement for the first time (see Figure 1E). Additional follow-ups confirmed tumor regression without any additional treatment (see Figure 1F).

An MRI 4.5 years after the SRT revealed an absence of any residual tumor and opening of the sylvian aqueduct (see Figure 1G). Informed consent was obtained from the patient for this study.

## Discussion

We present a case of significant enlargement of a low-grade glioma soon after SRT with subsequent complete regression of the tumor. The sylvian aqueduct block did not lead to occlusive hydrocephaly due to the previous ETV treatment. The stable state of the patient allowed us to continue observation and to register subsequent tumor regression without any additional treatment.

We regard the case as pseudoprogression. This term is usually used to describe the response to the irradiation and it means temporary tumor volume and contrast enhancement enlargement [1]. Pseudoprogression is well described in several tumors (vestibular schwannomas, malignant gliomas, and metastases).

However, such observations of pseudoprogression in patients with low-grade gliomas (LGG) are rarely published. One of the largest is the paper from Naftel, et al. who presented the results of the irradiation of 24 patients with LGG. Out of 13 patients with tumor volume enlargement, only two later confirmed tumor progression [2]. Mansur, et al. indicated that they have often registered reversible radiographic changes after radiotherapy for PA in their group of 35 patients [3].

Pseudoprogression should be distinguished from tumor growth. There is evidence that pseudoprogression is much more common after irradiation than true relapse [2]. If the patient's condition allows careful observation or conservative treatment, the evaluation of the dynamics in tumor size and contrast enhancement can reveal the nature of changes in the glioma.

Pseudoprogression with subsequent tumor regression should be differentiated from spontaneous tumor shrinkage which is described for PA. It appears that true incidence of spontaneous (without any treatment) tumor shrinkage is quite low [4-5]. The main part of the cases described in the literature [4-5] is associated with the methodological pitfalls of the studies. While spontaneous regression is usually registered after partial resection, pseudoprogression has a distinct time connection with irradiation.

## Conclusions

We regard this clinical case as an example of PA pseudoprogression following radiotherapy. Based on the findings of other authors and our data we can assume that the described tumor changes after irradiation of PA are as often pseudoprogression as in the case of vestibular schwannomas. A correct estimation of the situation is required to determine the proper treatment.
